# In memoriam: Gita Ramjee

**DOI:** 10.1002/jia2.25497

**Published:** 2020-05-07

**Authors:** Linda‐Gail Bekker, Gavin Churchyard, Lulu Nair, Glenda Gray

**Affiliations:** ^1^ Desmond Tutu HIV Centre University of Cape Town Cape Town South Africa; ^2^ The Aurum Institute Johannesburg South Africa; ^3^ Perinatal HIV Research Unit Soweto South Africa; ^4^ South African Medical Research Council South Africa

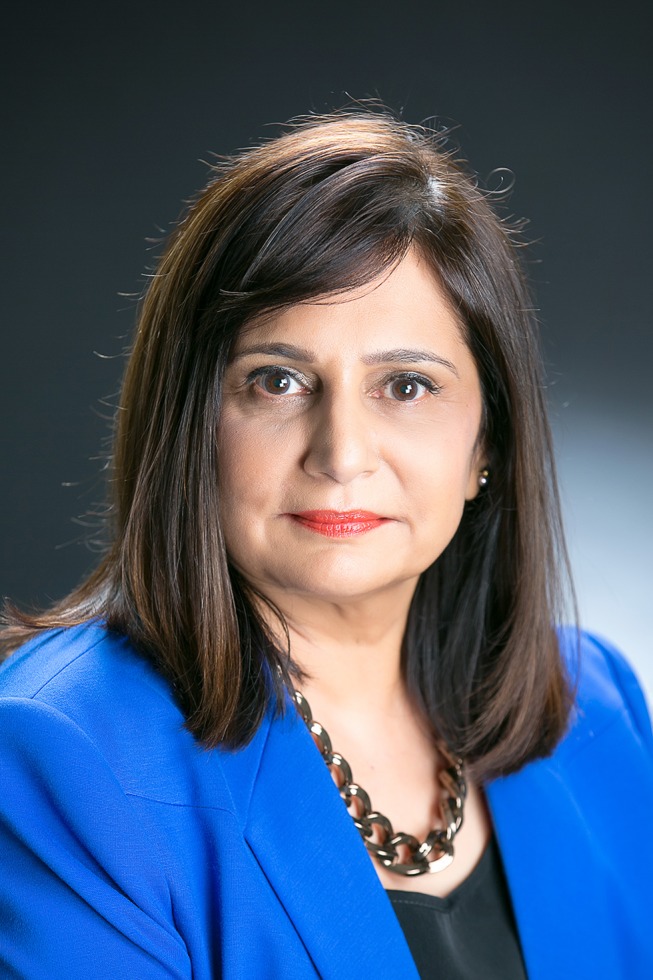

*“I learned about the dire need for women‐initiated HIV prevention options and the socio‐behavioural and cultural factors that impact women’s lives. I dedicated my time to researching methods of HIV prevention*.” – Dr. Gita Ramjee

The world has lost a fierce champion, strategic advocate and excellent researcher with the tragic and untimely passing of Prof. Gita Ramjee in Kwa Zulu Natal, South Africa on 31^st^ March 2020. Gita will be best remembered for her tireless commitment to finding workable and effective HIV prevention solutions for women that encompassed all known determinants and drivers of HIV, from the sociopolitical to the biobehavioural.

Qualifying as a basic scientist from the University of Sunderland, United Kingdom, Gita moved to South Africa and completed a Master’s degree in the role of aflatoxins in childhood malnutrition. Her PhD focused on the role of proteinuria in childhood kidney diseases. Thereafter her scientific work turned to the early HIV epidemic, where she led a project on vaginal microbicides for the prevention of HIV among a group of sex workers working along the trucking route between the port city of Durban and the commercial capital in Johannesburg. That study was pivotal in her career, as she became aware first hand of the dire need for women‐initiated HIV prevention options and the socio‐behavioural and cultural factors that impact women’s lives. This became her guiding principle and her passion.

Gita joined the Medical Research Council in 1994 as a senior researcher. During this period she was involved in numerous research projects and progressed to become the Divisional Head of the Centre for Epidemiological Studies in South Africa. In 2001, she was promoted to the position of Acting Director of the HIV Prevention Research Unit. Her vision was to develop a centre of excellence in HIV prevention. Through her strategic planning, the HIV Prevention Research in the Unit expanded to a multi‐disciplinary program of HIV Prevention, Treatment and Care. Gita was a study principal investigator and unit director for many of the US National Institutes of Allergy and Infectious Diseases and the Division of AIDS HIV prevention networks clinical trials, ranging from early phase studies to late phase efficacy trials. She contributed to our understanding of the role of topical and systemic products in HIV prevention as well as active and passive immunization research. She was instrumental in developing standards of care in clinical trials and her more recent work had expanded to integrate HIV/TB prevention and treatment.

She was the recipient of many national and international awards for her research including the “Outstanding Female Scientist” Award by the European Development Clinical Trials Partnerships (EDCTP) in Lisbon, Portugal, alongside other global academic giants. The Award was in recognition of her life’s work and dedication to finding new HIV prevention methods, which are conducive to the lifestyles and circumstances of women in South Africa. She received the Lifetime Achievement Award at the International Microbicide Conference in Sydney, Australia, in 2012 and the South African Medical Research Council Scientific Merit Award 2017 Gold Medal.

In her leadership role, she trained and mentored numerous cohorts of scientists and clinical trial staff, who now populate the network of trial sites in South Africa and beyond. They will carry her legacy forward for many years.

In her role as mentor, she said in 2019 when interviewed “Interest in science and an inquiring mind is critical. If you have these qualities and have the passion, tenacity and determination to pursue a career where you may not always get the desired answer but have the commitment to make a difference in the lives of people, albeit in a small way, then you should definitely pursue a science career. Love of the job, passion, drive and tenacity are critical traits to have for scientific excellence.”

After almost two decades at the South African Medical Research Council in Kwa Zulu Natal, Gita took on the position of Chief Scientific Officer, HIV, for the Aurum Institute in Johannesburg in 2019. After returning from delivering a paper in London, she contracted and sadly succumbed to complications associated with SARS‐CoV‐2 infection.

Gita is survived by her loving husband and two adult sons and their families. She was immensely proud of her family and their successes, and family life contributed to her sense of fulfilment and happiness.

Prof. Gita Ramjee will certainly be loved and remembered for her love of the job, her passion, drive and tenacity and the enormous contribution she has made to science in South Africa and globally. More than this, her contribution to women, their children and their partners, worldwide will live on.

## Competing interests

None.

## Authors’ contributions

All authors have contributed to the preparation of the manuscript, read and approved the final draft.

